# Six potential biomarkers in septic shock: a deep bioinformatics and prospective observational study

**DOI:** 10.3389/fimmu.2023.1184700

**Published:** 2023-06-08

**Authors:** Chang Kong, Yurun Zhu, Xiaofan Xie, Jiayu Wu, Meizi Qian

**Affiliations:** ^1^ Department of Anesthesiology and Critical Care Medicine, Tianjin Nankai Hospital, Tianjin Medical University, Tianjin, China; ^2^ Department of Anesthesia, The First Affiliated Hospital of Wenzhou Medical University, Wenzhou, Zhejiang, China; ^3^ Department of General Practice, Central Health Center of Yayang Town, Taishun County (Yayang Branch of Medical Community of Taishun County People’s Hospital), Wenzhou, Zhejiang, China; ^4^ Key Laboratory of Intelligent Treatment and Life Support for Critical Diseases of Zhejiang Province, Wenzhou Medical University, Wenzhou, China

**Keywords:** septic shock, WGCNA, biomarker, bioinformatic analysis, prognosis

## Abstract

**Background:**

Septic shock occurs when sepsis is related to severe hypotension and leads to a remarkable high number of deaths. The early diagnosis of septic shock is essential to reduce mortality. High-quality biomarkers can be objectively measured and evaluated as indicators to accurately predict disease diagnosis. However, single-gene prediction efficiency is inadequate; therefore, we identified a risk-score model based on gene signature to elevate predictive efficiency.

**Methods:**

The gene expression profiles of GSE33118 and GSE26440 were downloaded from the Gene Expression Omnibus (GEO) database. These two datasets were merged, and the differentially expressed genes (DEGs) were identified using the limma package in R software. Gene Ontology (GO) and Kyoto Encyclopedia of Genes and Genomes (KEGG) pathway enrichments of DEGs were performed. Subsequently, Lasso regression and Boruta feature selection algorithm were combined to identify the hub genes of septic shock. GSE9692 was then subjected to weighted gene co-expression network analysis (WGCNA) to identify the septic shock-related gene modules. Subsequently, the genes within such modules that matched with septic shock-related DEGs were identified as the hub genes of septic shock. To further understand the function and signaling pathways of hub genes, we performed gene set variation analysis (GSVA) and then used the CIBERSORT tool to analyze the immune cell infiltration pattern of diseases. The diagnostic value of hub genes in septic shock was determined using receiver operating characteristic (ROC) analysis and verified using quantitative PCR (qPCR) and Western blotting in our hospital patients with septic shock.

**Results:**

A total of 975 DEGs in the GSE33118 and GSE26440 databases were obtained, of which 30 DEGs were remarkably upregulated. With the use of Lasso regression and Boruta feature selection algorithm, six hub genes (*CD177*, *CLEC5A*, *CYSTM1*, *MCEMP1*, *MMP8*, and *RGL4*) with expression differences in septic shock were screened as potential diagnostic markers for septic shock among the significant DEGs and were further validated in the GSE9692 dataset. WGCNA was used to identify the co-expression modules and module–trait correlation. Enrichment analysis showed significant enrichment in the reactive oxygen species pathway, hypoxia, phosphatidylinositol 3-kinases (PI3K)/Protein Kinase B (AKT)/mammalian target of rapamycin (mTOR) signaling, nuclear factor-κβ/tumor necrosis factor alpha (NF-κβ/TNF-α), and interleukin-6 (IL-6)/Janus Kinase (JAK)/Signal Transducers and Activators of Transcription 3 (STAT3) signaling pathways. The receiver operating characteristic curve (ROC) of these signature genes was 0.938, 0.914, 0.939, 0.956, 0.932, and 0.914, respectively. In the immune cell infiltration analysis, the infiltration of M0 macrophages, activated mast cells, neutrophils, CD8 T cells, and naive B cells was more significant in the septic shock group. In addition, higher expression levels of *CD177, CLEC5A, CYSTM1, MCEMP1, MMP8*, and *RGL4* messenger RNA (mRNA) were observed in peripheral blood mononuclear cells (PBMCs) isolated from septic shock patients than from healthy donors. Higher expression levels of CD177 and MMP8 proteins were also observed in the PBMCs isolated from septic shock patients than from control participants.

**Conclusions:**

*CD177*, *CLEC5A*, *CYSTM1*, *MCEMP1*, *MMP8*, and *RGL4* were identified as hub genes, which were of considerable value in the early diagnosis of septic shock patients. These preliminary findings are of great significance for studying immune cell infiltration in the pathogenesis of septic shock, which should be further validated in clinical studies and basic studies.

## 1 Introduction

Sepsis is a life-threatening organ dysfunction disease that is characterized as an unusual systemic reaction to what is sometimes an otherwise ordinary infection (1). Septic shock is a subtype of sepsis with circulatory and cellular or metabolic dysfunction (2). Both sepsis and septic shock lead to high mortality and morbidity rates and represent a heavy societal burden across the world (3). By identifying the root causes and relevant diagnostic biomarkers of septic shock, we can intervene early and effectively, resulting in a significant reduction in the mortality rates associated with sepsis. Therefore, it is crucial to research the biological mechanisms involved in septic shock and explore the potential indicators for early diagnosis.

It is believed that immune cells and immune-related pathways significantly contribute to the development of septic shock; this is based on growing clinical and experimental evidence supporting the association between the overactivation of innate immune effector cells and uncontrolled inflammation leading to tissue damage and organ failure in severe septicemia observed in both human subjects and animal models (4–6). Judith Hellman et al. proposed that sepsis disrupts the balance of the redox state toward a pro-oxidative state. This is characterized by the excessive production of reactive oxygen and nitrogen species, mitochondrial dysfunction, and damage to the antioxidant system. It results in microvascular dysfunction and multiple organ failure (7). Research has shown that activating the phosphatidylinositol 3-kinases (PI3K)/AKT/mammalian target of rapamycin (mTOR) pathway and inhibiting the nuclear factor-κβ (NF-κβ) signaling pathway can have a protective effect on organs in the pathological and physiological state of sepsis (8).

To better understand the dysregulated immune responses to infection that are sepsis and septic shock, biomarkers have been evaluated for providing information beyond what is available using other metrics, and could therefore help inform clinical decision-making and potentially improve patient management (9). It has been reported that several proposed biomarkers, such as C-reactive protein (CRP) and procalcitonin (PCT), could complement clinical evaluation and help physicians to make therapeutic decisions (10); however, the role of biomarkers in septic shock diagnosis remains undefined (11). In addition, there are few prognosis specificity markers that have performed well in preventing septic development from reaching a severe phase (12–14). Therefore, this study aims to explore the value of immune-related genes in the early diagnosis of septic shock at the genetic level. A better understanding of the role of immune regulatory genes in promoting the diagnosis of septic shock is of great significance and can provide new ideas for the accurate diagnosis and treatment of septic shock.

In this study, we conducted a comprehensive bioinformatics analysis based on a large sample size to screen for promising gene markers for septic shock. The microarray datasets for septic shock were downloaded from the Gene Expression Omnibus (GEO) database. Subsequently, differentially expressed genes (DEGs) analyses were performed for both the patients with septic shock and control participants. In sepsis, genes play their role through networks of co-expression genes with alike biological roles. The recognition of co-expressing patterns can offer more inspiration on septic pathways. Therefore, we also analyzed the relationship between gene expression and phenotypes *via* weighted gene co-expression network analysis (WGCNA) in septic shock (15, 16). Then, hub genes screened from DEGs for septic shock and those identified using WGCNA were intersected and analyzed. Functional and pathway analyses were also performed, and the relationships of proteins were investigated.

The infiltration of inflammatory cells and activation of immune-related pathways are critical features in sepsis and septic shock (17, 18). In addition, inflammatory cytokines such as interleukin-1β (IL-1β), IL-6, IL-18, tumor necrosis factor alpha (TNF-α), and interferon-gamma (IFN-γ) have been reported to play an essential role in the development of sepsis and septic shock (19, 20). However, the changes in immune cell types in response to septic shock are still unclear. The critical role of particular immune cells and immune-related pathways in septic shock was confirmed by these molecular and cellular experiments, but few studies have been conducted that explore the correlation of genes and immune cells or the overall landscape in big data. Hence, we conducted an overall description of the immune-related landscape of septic shock in this study. Finally, the diagnostic values of hub genes were evaluated by ROC analyses and the measurement of mRNA concentration and protein expression levels, and we also conducted a preliminary verification of data credibility.

The results given in the present study could be conducive to comprehensively understanding septic shock pathogenesis, identifying the molecular mechanisms that are involved in the pathological process, and providing insights into novel treatment and therapeutic targets for drugs.

## 2 Materials and methods

### 2.1 Extraction of peripheral blood mononuclear cells from clinical blood samples

Our study is a forward-looking, observation-originated investigation involving septic patients from the intensive care unit (ICU) of the First Affiliated Hospital of Wenzhou Medical University from June 2021 to November 2021. All patients in our study had reached the consentaneous standards of the Third International Consensus Definitions for Sepsis and Septic Shock (Sepsis-3) (11). Septic shock patients exhibited persistently low blood pressure, which required vasopressors for the maintenance of MAP ≥ 65 mmHg and sera lactic acid levels > 2 mmol/L (18 mg/dL) despite sufficient fluid resuscitation. Patients who were < 18 years old or who had potential acute non-infection organic damage and shock were filtered out.

Blood samples were collected in ethylenediaminetetraacetic acid (EDTA) tubes immediately upon patient admission, before septic shock occurred. Blood samples from 30 control participants were also collected, followed by the immediate extraction of peripheral blood mononuclear cells (PBMCs). The isolation of PBMCs from whole blood is based on the density differences between PBMCs and other components of the peripheral blood. The origins of this method go back to the 1960s, when Arne Boyum first described the separation of white blood cells using Ficoll and other density gradient media. Since then, the technique has been refined, but the underlying principles have remained the same: by density gradient centrifugation, separation of blood components according to their density is possible with the help of a density gradient medium (e.g., Lymphoprep or Ficoll) containing sodium diatrizoate, polysaccharides, and water, reaching a density of 1,077 g/mL. The density gradient medium facilitates the aggregation of red blood cells. The medium is denser than lymphocytes, monocytes, and platelets, but less dense than granulocytes and red blood cells. Accordingly, erythrocytes and most of the granulocytes will sediment and pellet at the bottom of the tube after centrifugation; after this phase, the density gradient medium is found. The top layer consists of plasma and platelets. Mononuclear cells band at the interface between the plasma and the density gradient medium. Subsequently, two washing steps at a lower speed help to remove the remaining platelets (21). The PBMCs obtained were stored at –80°C for subsequent experimental research.

### 2.2 Real-time quantitative PCR

The RNA from cells was extracted using Trizol (Invitrogen) and reverse transcribed into cDNA through a synthesis kit containing specific primers and SYBR green reaction mix (Takara Clontech, Dalian, Japan). Specific primers were customized by Sangon Biotechnology Co., Ltd. (Shanghai, China), with the presented sequence shown in **Table 1**. Real-time qPCR was performed, followed by the calculation of relative gene expression levels using the 2^–ΔΔCT^ approach. Each primer sequence is presented in **Table 1**.

**Table 1:** Primers used for qPCR of genes from human.

Gene	Forward (5′-3′)	Reverse (5′-3′)
*CYSTM1*	CTTATCCACCACAACCAATGGG	GGATGGTCCTAGCTCATCTCTT
*CLEC5A*	AGGTGGCGTTGGATCAACAA	TTAGGCCAATGGTCGCACAG
*CD177*	ATGAGCGCGGTATTACTGCTG	GGTCGGACACCTTCCACAC
*MCEMP1*	CCATGCAAAGGGTGGTCATTC	GCTTGTACGGAGTTTGAGACATT
*MMP8*	TGCTCTTACTCCATGTGCAGA	TCCAGGTAGTCCTGAACAGTTT
*RGL4*	CTGGGCAACACGCATTAACAA	GTTCTTTACAGACCCACGACAG
*GAPDH*	GGAGCGAGATCCCTCCAAAAT	GGCTGTTGTCATACTTCTCATGG

qPCR, quantitative real-time polymerase chain reaction.

### 2.3 Western blotting

Proteins were extracted from PBMCs, after which the protein concentration was measured using a bicinchoninic acid (BCA) protein assay (Beyotime, P0012, Shanghai, China). An equal number of proteins (20 μg) per sample was separated by 10% sodium dodecyl sulphate-polyacrylamide gel electrophoresis (SDS-PAGE), then transferred to a Polyvinylidene difluoride (PVDF) membrane (Millipore). Subsequently, the membranes were incubated with anti-CD177 (HUABIO, ER65526, 1:1,000) and anti-MPP8 (HUABIO, ER63980, 1:1,000) overnight after being blocked, firstly by 5% skim milk at room temperature for 2 h, and thereafter by a horseradish peroxidase (HRP)-conjugated secondary antibody. The images were scanned and quantified using Image Lab software (Bio-Rad).

### 2.4 Data collection and ethics statement

Relative clinical information was documented, including patient demographics and diagnostic results, and also their PCT, lactic acid, and CRP levels. Acute Physiology and Chronic Health Evaluation (APACHE) II scores were recorded at the onset of septic shock and sequential organ failure assessment (SOFA) scores were recorded in the first 24 hours after the diagnosis of septic shock. Clinical test results and hospital death rate data were also collected within the first 24 hours of ICU admission. Our research (clinical trial registration number: ChiCTR2100053564) was approved by the ethics committee of the First Affiliated Hospital of Wenzhou Medical University, Zhejiang, China, and recorded at ClinicalTrials.gov., in accordance with the protocols in the Declaration of Helsinki, with informed consent provided by each participating patient.

### 2.5 Microarray data

The gene expression profiles of GSE33118, GSE26440 (22), and GSE9692 (23) were downloaded from the GEO database (http://www.ncbi.nlm.nih.gov/geo/) (24). The GSE33118 data collection includes the gene expression profiles of 20 septic shock patients and 42 control participants; blood specimens were obtained within 24 h of the septic shock diagnosis. The samples in the GSE26440 data collection were for 98 children with septic shock and 32 control pediatric patients. Children who were 10 years old in the pediatric intensive care unit (PICU) and who met the pediatrics-specific standards for septic shock were enrolled (25). After obtaining written informed consent, blood specimens were acquired within 24 h of their initial presentation to the PICU. The specimens in the GSE9692 data collection were also collected; these were for 42 septic shock patients who were minors, and 15 healthy donors. The patients who had one or more serious coexisting diseases or who were undergoing immunosuppression treatment were filtered out. The septic shock diagnoses were referred to the American College of Chest Physicians/Society of Critical Care Medicine (ACCP/SCCM) standards.

### 2.6 Identification of DEGs

We downloaded the GSE33118 datasets, which contained the expression profiles of 150 groups of patients, and GSE26440, which contained the expression profiles of the normal group (*n* = 42) and the disease group (*n* = 108) that were related to septic shock from the GEO database. To obtain the critical information from the two datasets, the merging and pre-processing of raw data were conducted using the surrogate variable analysis (SVA) package (26) from R software. Principal component analysis (PCA), a multivariate regression analysis, was used to distinguish samples with multiple measurements (27, 28). We used PCA to show the uniformity of the two datasets after correction. In addition, the limma package (29) was used to conduct a differential analysis of the combined database comprising these two databases; gene screening conditions with *p* < 0.05 and | Log2FC | > 1 were used as the selection criteria for filtering septic shock DEGs.

### 2.7 Functional enrichment analysis of DEGs and PPI network construction

Gene Ontology (GO), a major bioinformatics tool for annotating genes and analyzing biological process, and Kyoto Gene Genome Encyclopedia (KEGG), a database resource for understanding high-level functions and biological systems from large-scale molecular datasets generated by high-throughput experimental technologies, were used to further investigate the biological functions and signaling pathways involved in the occurrence and development of septic shock. The protein–protein interaction (PPI) networks of the septic shock DEGs were predicted through the retrieval pool Search Tool for the Retrieval of Interacting Genes/Proteins (STRING). The statistical significance was set at a minimum overlap of ≥3 and *p *≤ 0.01. Afterward, the Cytoscape program was used to establish and realize the visualization of molecule mutual effect nets (30). In the PPI network, the target proteins were represented by nodes, whereas the predicted or validated interactions between proteins were represented by edges.

### 2.8 Lasso regression and Boruta feature selection process

Lasso logistic regression was used for feature selection of the diagnostic markers of disease. The Lasso algorithm uses the generalized linear model net (GLMNet) software package (version 4.1–2). In addition, Boruta is a feature selection algorithm that randomly disrupts each real feature in order, evaluates the importance of each feature, and iteratively removes features with low correlation to find the best variable. In this study, the Boruta package (version 7.0.0) was used for feature selection, and 500 trees were constructed to further identify the diagnostic value of these biomarkers for disease.

### 2.9 WGCNA analysis

Through the establishment of a weight-added genetic co-expressing net, our team searched for co-expressed genetic modules and explored the correlation between gene networks and phenotypes, along with the core genes (15). The WGCNA-R packet (version 1.70–3) was adopted to establish the co-expression net of all genes in the dataset, and the genes with the top 5,000 variance were selected *via* such arithmetic for further assays, in which the soft liminal value was 14. The weight-added adjacent matrix was converted into a topological overlap matrix (TOM) to speculate the net connection level. The adjacency matrix is a matrix composed of weighted correlation values between genes, whereas TOM is a new distance matrix that is transformed by the adjacency matrix to reduce noise and false correlation. The topological overlap matrix considers not only the direct relationship between genes, but also the possible multiple indirect relationships. This information can be used to construct a network for subsequent analysis.

The hierarchy clustering approach was adopted to establish the cluster tree framework of TOM matrix. Diverse offshoots of the cluster tree represent diverse genetic modules, and the diverse colors denote diverse modules. As per the weight-added association coefficient of genes, the genes were categorized in terms of their expressing features. Genes with similar features were classified into a module, and massive genes were divided into multi-modules *via* genetic expressing features. After genes were clustered, a heatmap was plotted to visualize and calculate the intermodule correlation. The correlation between modules and clinical traits was further evaluated to determine the modules associated with septic shock for analysis.

### 2.10 Veen and circos analysis

The Venn diagram, which is a widely used diagram that exhibits the relationship between multiple sets, is probably the most intuitive form of data visualization, superior to heat maps and tables when the number of sets is fewer than five (31). In addition, we used the Circlize and Corrplot packages to plot the interaction diagram of core genes expressing positive and negative correlation.

### 2.11 GeneMANIA analysis

GeneMANIA is an elastic, easy-to-use PPI net construction data center for the visualization of function networks between genes and the analysis of gene roles and mutual effects. The database can set up the data resource of genetic nodal points and has various biological information assay approaches, such as physical mutual effect, genetic co-expression, genetic co-location, genetic enriching assay, and database forecast. In this study, the core gene net was generated using GeneMANIA to explore its possible mechanism in septic shock.

### 2.12 Gene set difference analysis

Gene set difference analysis (GSVA) is a non-parameter, non-supervised approach for assessing the enrichment of transcriptomic genomes. Through the all-round scoring of the intriguing genetic sets, GSVA transforms the genetic-level variations into pathway-level variations and afterwards judges the biology role of the samples. The genetic sets used in this study were acquired from Molecular Signatures Data Center 7.0, and the GSVA arithmetic was adopted for the all-round scoring of every genetic set to assess the underlying biological function variations of diverse specimens.

### 2.13 Receiver operating characteristic curve analysis

MedCalc statistical software (www.medcalc.org) was used to carry out receiver operating characteristic (ROC) curve assay and to identify the idiosyncrasy, susceptibility, probability ratio, and favorable and unfavorable prediction scores for all probable liminal values of the ROC curve. The genetic scores were forecasted as per the ROC curve assay.

### 2.14 CIBERSORT analysis of immune infiltration

CIBERSORT algorithm (32), which is a gene expression-based arithmetic that represents cell composition based on pre-processed gene expression profiles, was utilized to evaluate immune cell infiltration in septic shock. LM22 (22 immune cell types) in CIBERSORT is a signature gene expression matrix used to estimate the proportion of leukocytes. Data from control participants and patients with septic shock were analyzed using the CIBERSORT algorithm to infer the relative proportions of 22 immune infiltrating cells, with the sum of all estimated immune cell type scores in each sample equal to 1. In this study, the influence of genes on immune infiltration was evaluated. CIBERSORT (http://CIBERSORT.stanford.edu/) was used to quantify the level of immune cell infiltration in each sample, and Spearman’s correlation analysis was performed on gene expression level and immune cell content.

### 2.15 Statistical analysis

Our team undertook statistic assays *via* GraphPad Prism 8, SPSS 21.0, and R program 4.0. The normal distribution successive variates were expressed as averages ± SD and were studied *via* an independent Student’s *t*-test or one-way ANOVA. *P-*values < 0.05 were deemed to be statistically significant.

## 3 Manuscript formatting

### 3.1 Identification of DEGs, functional enrichment analysis, and construction of PPI networks

**Figure 1:**
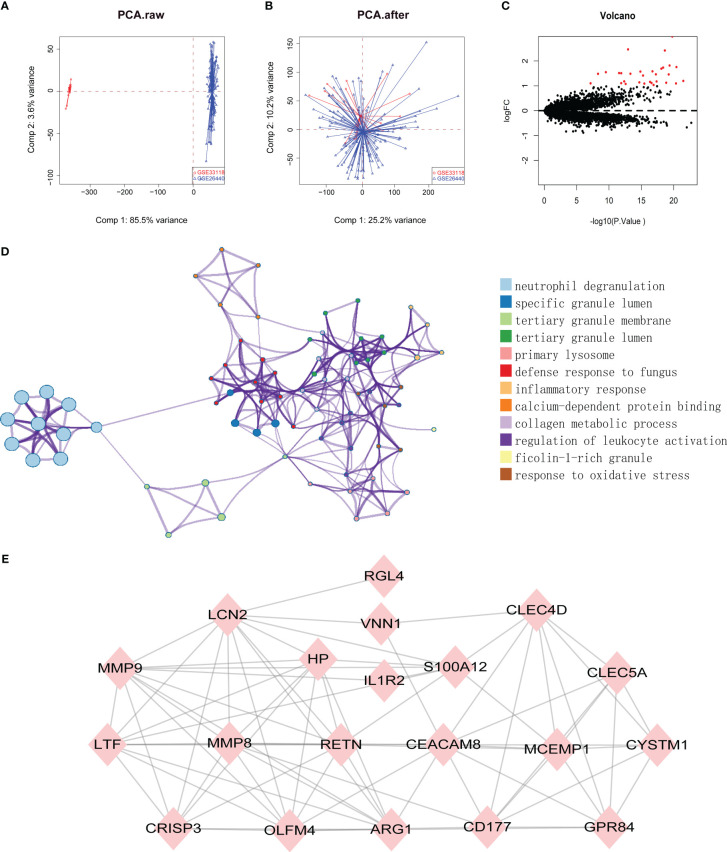
**(A, B)** SVA algorithm was used to correct the chip, and PCA (Principal Component Analysis) diagram was used to show the results after correction. The results showed that the interchip batch effect was eliminated after SVA algorithm correction. **(C)** Volcano plot of gene expression profile data between normal group and septic shock group. Red dots: significantly up-regulated genes; Black dots: nondifferentially expressed genes. P<0.05 and |log2 Foldchange |>1 were considered as significant. **(D)** The classification netplot denoted the associations of genes and GO terms as a net to indicate the enriched pathways, including neutrophil degranulation, SGL, TGL, and primary Lysosome and other pathways. **(E)** The PPI networks based on DEGs involved 30 key genes.

Datasets related to septic shock were downloaded from the GEO database, then the GSE33118 and GSE26440 datasets were merged. A total of 150 gene expression profiles were included, containing 108 septic shock patients and 42 controls participants. The batch effect was eliminated by sva package, which is presented in a principal component analysis (PCA) diagram (**Figures 1A, B**). In total, 975 differentially expressed genes (DEGs) were identified by screening *via* the limma package in R software, of which 30 were upregulated genes (**Figure 1C**, red dots) (*p *< 0.05).

Subsequently, GO functional enrichment analysis and KEGG pathway analysis were conducted, suggesting that neutrophil degranulation, specific granule lumen, tertiary granule membrane, tertiary granule lumen, and primary lysosome and other pathways may play important roles in septic shock (**Figure 1D**). Using the PPI network, 30 genetic variations that may be associated with septic shock were identified. They were *CYSTM1*, *MCEMP1*, *GYG1*, *MMP8*, *HP*,*HPR*, *GPR84*, *RGL4*, *CD177*, *RETN*, *BMX*, *CLEC5A*, *CA4*, *IL1R2*, *S100A12*, *ARG1*, *PFKFB3*, *ANKRD22*, *ANXA3*, *CLEC4D*, *MMP9*, *OLFM4*, *MS4A4A*, *VNN1*, *LCN2*, *TDRD9*, *LTF*, *OLAH*, *CEACAM8*, and *CRISP3*. These are shown in **Figure 1E**.

### 3.2 Identification of hub genes

**Figure 2:**
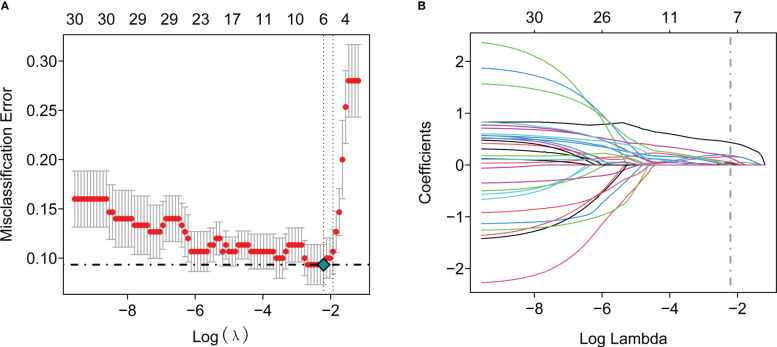
The Lasso regression and Boruta feature selection algorithm were used to screen out the characteristic genes in septic shock. **(A)** 6 differential genes were identified as key genes of septic shock by Lasso regression. **(B)** 24 differential genes were identified as key genes of septic shock by Boruta algorithm.

To further identify the hub genes among the differential genes, we combined Lasso regression and Boruta feature selection algorithm to screen out the characteristic genes of septic shock patients. The results of Lasso regression showed that six differential genes were identified as hub genes of septic shock (**Figure 2A**, **Supplementary Table 1**), 24 differential genes were identified as the hub genes of septic shock by Boruta algorithm (**Figure 2B**, **Supplementary Table 2**), and six hub genes were screened out after the two were intersected (**Table 2**): the CD177 molecule (*CD177*), C-type lectin domain containing 5A (*CLEC5A*), cysteine rich transmembrane module containing 1 (*CYSTM1*), mast cell expressed membrane protein 1 (*MCEMP1*), matrix metallopeptidase 8 (MMP8), and ral guanine nucleotide dissociation stimulator like 4 (*RGL4*).

**Table 2:** The six genes screened out by Lasso regression and Boruta selection algorithm in septic shock.

Gene	Description	Expr	Adjusted *p*-value	Protein function
*CYSTM1*	Cysteine rich transmembrane module containing 1	2.7	2.51E-18	Neutrophil degranulation
*CLEC5A*	C-type lectin domain containing 5A	2.7	4.55E-15	Regulates inflammatory responses
*CD177*	CD177 molecule	4.1	3.38E-16	Degranulation and superoxide production
*MCEMP1*	Mast cell expressed membrane protein 1	3.4	2.04E–17	Neutrophil degranulation
*MMP8*	Matrix metallopeptidase 8	4.5	6.36E-17	Degrade fibrillar type I, II, and III collagens
*RGL4*	Ral guanine nucleotide dissociation stimulator like 4	2.5	2.24E-16	Small GTPase mediated signal transduction

### 3.3 Construction of co-expression modules and module–trait correlation

**Figure 3:**
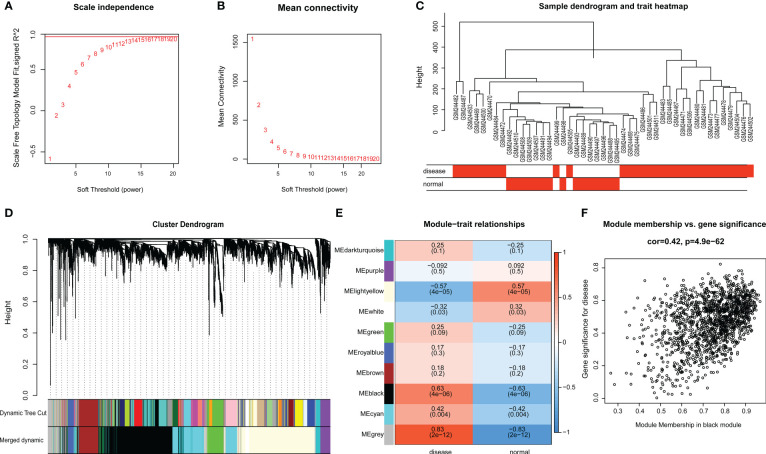
**(A, B)** Scale-free network construction in the co-expression network. Scale independence and mean connectivity analysis (β = 14). **(C)** Clustering tree construction. Merging of modules with similar expression profiles. **(D)** Module identification. Network dendrogram based on differential measurement and identified module colors, each node represents a gene, and the vertical axis denotes the degree of topological differences between genes, i.e. a larger distance between vertical coordinates indicates a larger topological difference, meaning a weaker co-expression correlation; the horizontal axes represent different modules, with each color denoting a module and the width of color bar indicating the gene number in the module; Dynamic Tree Cut represents modules initially obtained through average-linkage hierarchical clustering. Merged colors denote the reconstructed modules after merging similar modules. **(E)** Correlation of module and trait. Heatmap for the correlation between modules and septic shock traits. The horizontal axes represent different modules. The color of each cell indicates the corresponding module–trait correlation, a deeper red color suggesting stronger positive correlation and a deeper blue color suggesting stronger negative correlation. The value in each cell denotes the correlation score, and the value in the bracket below denotes the significance (*P*-value). **(F)** The association between modules and disease phenotype, the scatter plot of module eigengenes in the black module (COR = 0.42, P = 4.9E-62).

Raw microarray data from GSE9692 were used to construct the gene co-expression networks through WGCNA package to prune the gene modules of co-expression and explore the relationship between gene network and clinic features in the network. In samples of control and septic shock groups, there were no outliers detected in the expression matrix of the top 5,000 genes, whose expression variance was within the first quartile. To construct the correlation matrix, the Pearson correlation coefficient was utilized to calculate the correlation coefficient of expression between genes. An appropriate soft-thresholding power β = 14 was determined in the WGCNA package; here, the soft thresholding power is based on two criteria: the lowest power at which the scale-free topology fit index reached 0.90; and the connectivity measurements decrease considerably (**Figures 3A, B**).

The topological overlap matrix (TOM) was constructed from the adjacency matrix and used to cluster genes with high topological overlap into modules. The clustering dendrogram was then pruned using the dynamic tree cut method to further classify the modules (**Figure 3C**). However, we considered that some genes with high similarity were not clustered together, owing to the number of initially classified modules being relatively large. To avoid excessive module numbers that would complicate subsequent analysis, the module eigengenes in each module were clustered using a similarity threshold of 0.25 to group similar modules together. Finally, gene modules were detected based on TOM matrix, and 10 genetic modules were identified herein. They were the black (1426), brown (348), cyan (792), dark-turquoise (94), green (317), gray (283), light-yellow (1394), purple (166), royal-blue (108), and white (72) modules (**Figure 3D**).

The module–trait relationship heatmap (**Figure 3E**) was generated by analyzing the correlation between the constructed modules and traits and calculating their significance. The BLACK modules were found to be significantly associated with disease traits, indicating their role in the pathogenesis and progression of septic shock. Through further analysis of the association between modules and features, we discovered that the BLACK module exhibited the highest correlation with disease phenotype (COR = 0.42, *p* = 4.9E-62) (**Figure 3F**). Therefore, the BLACK module was selected for the subsequent analysis as septic shock-correlated modules.

### 3.4 Functional classification and pathway enrichment of hub genes

**Figure 4:**
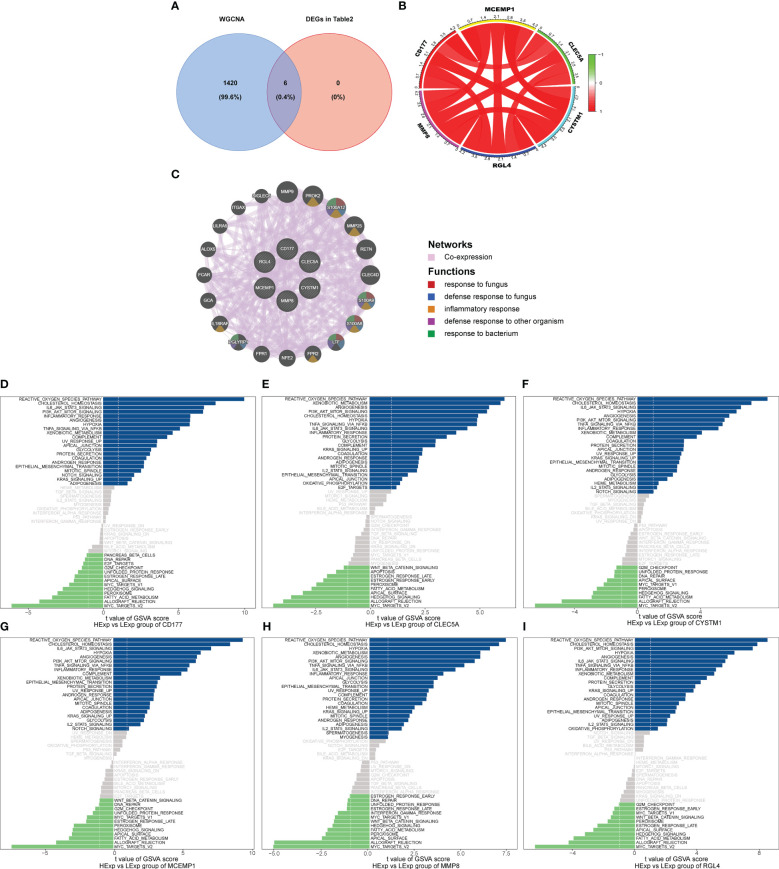
**(A)** Venn analysis for overlap of BLACK module target genes and six key DEGs (Identified from the intersection of Lasso regression and Boruta feature selection algorithm). **(B)** The interaction of positive and negative core genes was identified by circlize package and corrplot package. **(C)** The core gene net was generated through GeneMANIA to explore functions, including response to fungus, defense response to bacterium, inflammatory response, defense response to other organism and response to bacterium. The GSVA arithmetic was adopted for the all-round scoring of every genetic set, **(D)** CD177, **(E)** CLEC5A, **(F)** CYSTM1, **(G)** MCEMP1, **(H)** MMP8 and **(I)** RGL4 to assess the underlying molecule mechanisms where the key genes affect the development of septic shock, such as the reactive oxygen species pathway, hypoxia, PI3K/AKT/mTOR signaling, NFK-β/TNFa and IL6/JAK/STAT3.

We used the six hub genes that were identified as hub genes (shown in **Table 2**) to intersect with the BLACK module and found that all six hub genes intersected with the BLACK module, indicating that the results were in line with expectations (**Figure 4A**).

Subsequently, we used the “circlize” package and the “corrplot” package to plot the interaction of positive and negative core genes. Green represents a negative correlation and red represents a positive correlation. We found that *CD177*, *CLEC5A*, *CYSTM1*, *MCEMP1*, *MMP8*, and *RGL4* exhibited significant positive correlation with each other (**Figure 4B**). As shown in **Figure 4C**, the core gene net was generated through GeneMANIA to explore its possible mechanism in the disease, such as the response to fungus, defense response to bacterium, inflammatory response, and defense response to another organism.

Finally, we explored the specificity signal paths participating in the six hub genes to unveil the underlying molecule mechanisms where the hub genes impact the development of septic shock. GSVA then was used to determine the pathways in the following six genes: *CD177*, *CLEC5A*, *CYSTM1*, *MCEMP1*, *MMP8*, and *RGL4*. Among the abundant pathways involved in the six genes, we found several correlation pathways related to reactive oxygen species, namely hypoxia, PI3K/AKT/mTOR signaling, NFκ-β/TNFα, and IL6/JAK/STAT3, suggesting that the hub genes influence the development of septic shock by regulating these pathways (**Figures 4D–I**).

### 3.5 ROC analyses of biomarker genes

**Figure 5:**
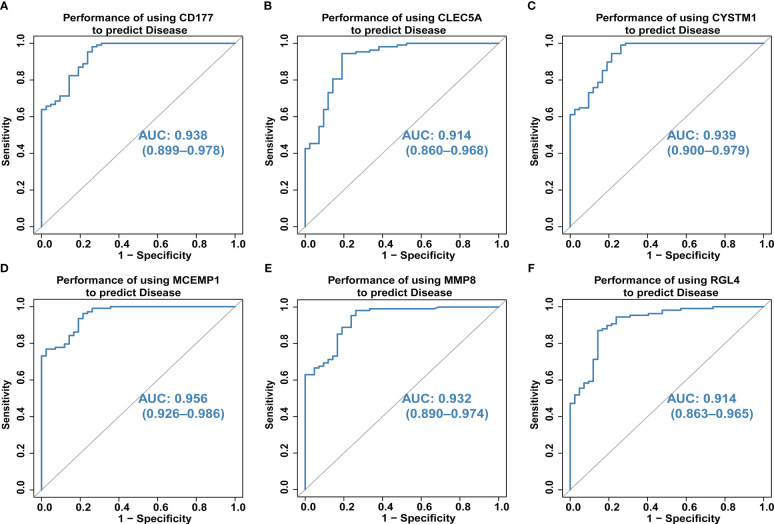
**(A–F)** The predictive ability of hub genes (CD177, CLEC5A, CYSTM1, MCEMP1, MMP8 and RGL4) on the occurrence and development of septic shock was explored through the ROC curve. The higher the AUC value, the better the predictive ability. The AUC for all six genes was >0.9 for ROC analysis.

We also explored the predictive ability of hub genes on the occurrence and development of septic shock through the ROC curve of diagnostic efficacy verification. The higher the AUC value, the better the predictive ability. The results showed that the AUC values of the six core genes were *CD177*-AUC: 0.938 (0.899–0.978), *CLEC5A*-AUC: 0.914 (0.860–0.968), *CYSTM1*-AUC: 0.939 (0.900–0.979), *MCEMP1*-AUC: 0.956 (0.926–0.986), *MMP8*-AUC: 0.932 (0.890–0.974), and *RGL4*-AUC: 0.914 (0.863–0.965) (**Figures 5A–F**).

### 3.6 Analysis of immune cell infiltration

**Figure 6:**
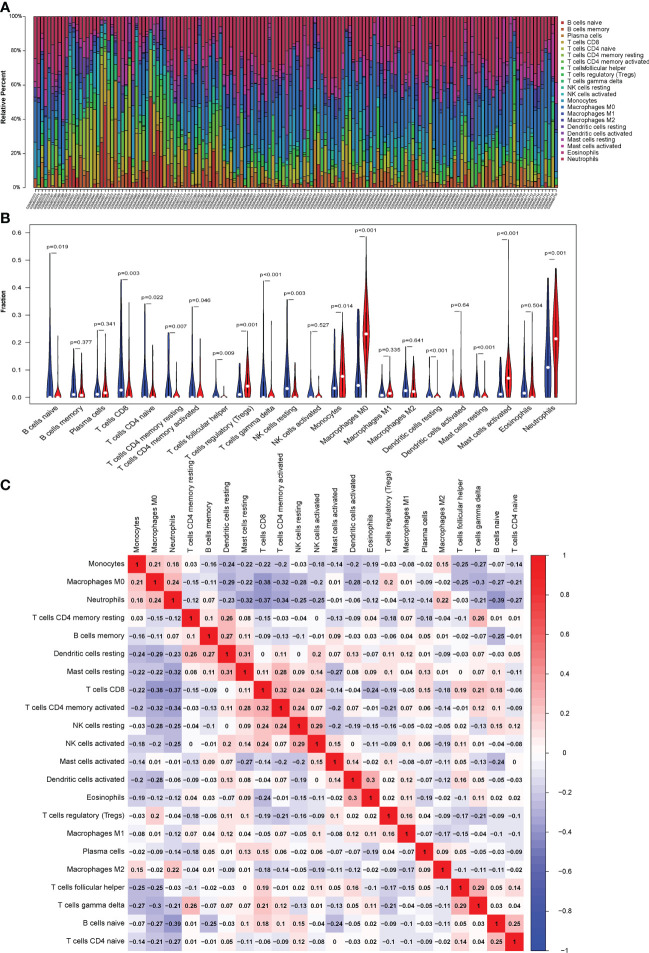
**(A)** CIBERSORT was used to quantify the fraction of 22 subsets of immune cells in septic shock. X axis: each GEO sample; Y axis: percentage of each kind of immune cells. **(B)** The violin graph shows the difference of immune infiltration between normal individuals and septic shock sufferers. The normal group is shown in blue and septic shock group is shown in red. P-Value < 0.05. **(C)** The co-expression patterns among fractions of immune cells. Red: positive correlation; blue: negative correlation.

The CIBERSORT algorithm was used to evaluate the proportion of different infiltrating immune cell types in the control and septic shock groups. The GEO expression array data were used to investigate the fraction of infiltrated immune cells. From 150 samples, 42 cases from the healthy donors group and 108 cases from septic shock group were eligible for CIBERSORT (*p*-value < 0.05). As shown in **Figure 6A**, the fraction of immune cells varied significantly among the samples and groups. The top five highest-infiltrating fractions in septic shock were M0 macrophages, activated mast cells, neutrophils, CD8^+^T cells, and I B cells. Follicular helper T cells and resting dendritic cells were present in lower quantities, inversely. Moreover, in general, higher proportions of M0 macrophages, activated mast cells, and neutrophils (*p*-value < 0.001) were found in the tissue of septic shock patients than in that of the healthy donors. In addition, we found a lower proportion of CD8^+^ T ceInaiveIlls, naive CD4^+^ T cells, gamma delta T cells, and resting natural killer (NK) cells in the tissue of septic shock patients than in that of the healthy donors (*p*-value < 0.05) (**Figure 6B**).

We conducted a correlation analysis of infiltrated immune cells in septic shock and found multiple pairs of positively and negatively related immune cells (**Figure 6C**). The score represents the degree of correlation. CD8^+^ T cells and activated CD4^+^ memory T cells showed the most synergistic effIand naive B cells and neutrophils showed the most competitive effect.

### 3.7 Hub genes and immune infiltration

**Figure 7:**
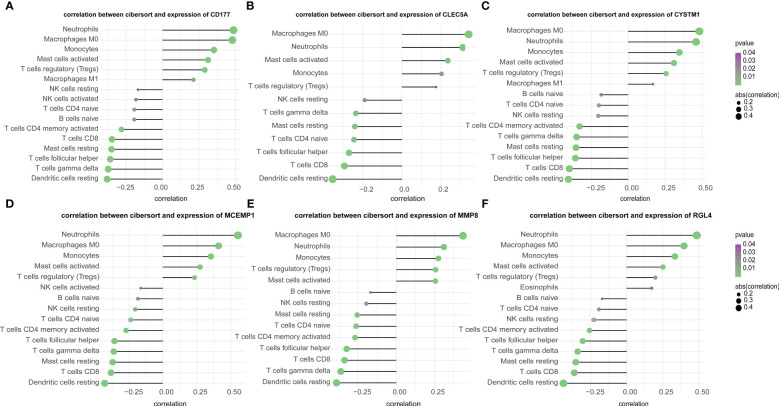
**(A–F)** Correlation of immune cells and 6 key genes. The all 6 genes **(A)** CD177, **(B)** CLEC5A, **(C)** CYSTM1, **(D)** MCEMP1, **(E)** MMP8 and **(F)** RGL4 were potently related to the similar kind of immunocyte content.

In this study, we analyzed the association between hub genes and immunity invasion in the septic shock dataset, so that the underlying molecular mechanism of hub genes affecting the development of septic shock was further explored. Correlation analysis with immune cells showed that all six hub genes were related to immune cells. In addition, the results in **Figures 7A–F** show that all six genes were positively related to M0 macrophages and neutrophils (*p*-value < 0.01) and negatively related to resting dendritic cells (*p*-value < 0.01).

The microenvironment primarily comprises immunocytes, the extracellular matrix (ECM), various growing factors, inflammation factors, and specific physicochemical properties, all of which remarkably influence the diagnosis and susceptibility of clinical treatment of diseases. Therefore, we further supplemented the correlation analysis to explore the relationship between the six hub genes and immunomodulatory genes (extended data are in **Supplementary Figures 1A–D**). From the an integrated repository portal for tumor-immune system interactions (TISIDB) database of immune activation genes, immunosuppression, chemokines, and chemokine receptors, we extracted four types of immune-regulatory genes; of these, six hub genes were found by correlation analysis to be significantly associated with immune activation (i.e., *CD27*, *CD68*, *ENTPD1*, *ICOS*, *TMEM173*), immunosuppression (i.e., *BTLA*, *CD244*, *CD274*, *HAVCR2*, *TGFB1*), chemokines (i.e., *CCL20*, *CXCL1*, *CXCL10*, *CXCL16*.) and chemokine receptors (i.e., *CCR1*, *CCR6*, *CXCR1*, *CXCR3*).

According to those comprehensive bioinformatics analyses, the aforementioned six hub genes may be considered new septic shock biomarkers, and further research is needed to verify these preliminary findings.

### 3.8 Preliminary validation analysis of biomarker genes

**Figure 8:**
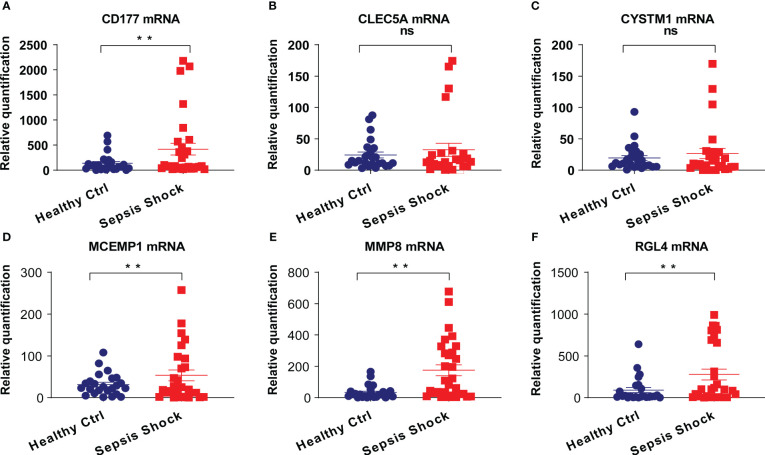
**(A–F)** The mRNA expression of CD177, CLEC5A, CYSTM1, MCEMP1, MMP8 and RGL4 were identified in PBMCs of sepsis sufferers and normal individuals via qRT-PCR. **P<0.05 vs relevant normal individuals; ns, no significance.

**Figure 9:**
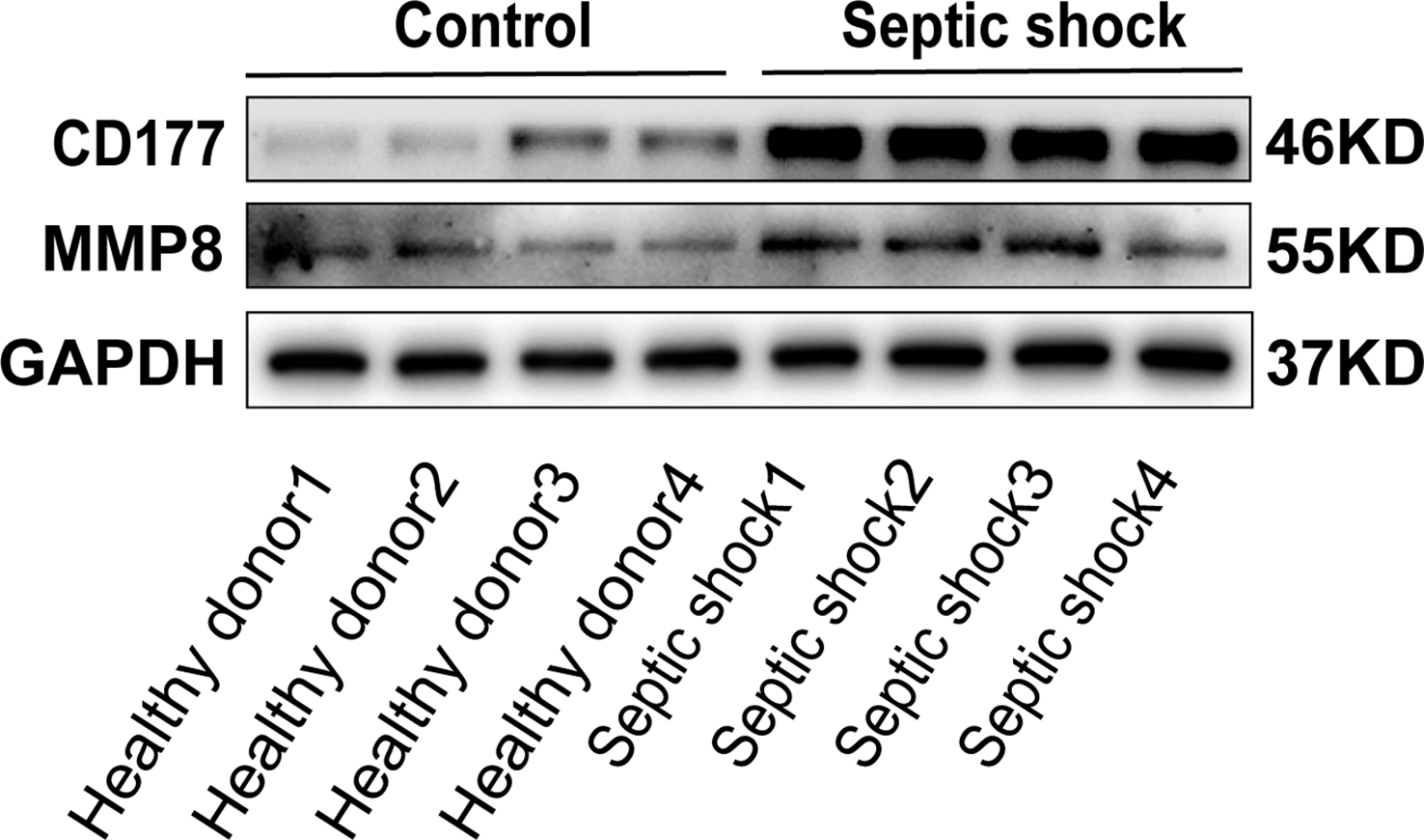
The protein expression of CD177 and MMP8 were identified in PBMCs of sepsis sufferers and health donors via Western blot.

To determine the clinical correlation of the discoveries made in this study, we undertook an RT-PCR analysis of peripheral blood mononuclear cells (PBMCs) from 30 septic shock patients and 28 healthy donors. The basic features are presented in **Table 3**. The age and sex distributional results had no statistical differences between these two groups. The most common infectious site was the lung (46.6%), then the abdomen and other organs (20.0%). Our studies revealed that the septic shock group displayed a greater total length of hospital stay and ICU stay in contrast to the healthy donors (*p* < 0.005). The APACHE II and SOFA values of the septic shock group were remarkably elevated in contrast to the healthy donors (*p* < 0.0001). The mRNA levels of *CD177*, *CLEC5A*, *CYSTM1*, *MCEMP1*, *MMP8*, and *RGL4* were remarkably incremented in the septic shock group (*n* = 30) compared with the control group (*n* = 28) (**Figures 8A–F**). Meanwhile, the expression levels of *CD177* and *MMP8*, as determined by Western blotting, were found to be upregulated in the PBMCs from septic shock patients (*n *= 4) compared with those from healthy donors (*n *= 4) (**Figure 9**). We found that these data exhibited a consistent trend with the bioinformatics analysis.

**Table 3:** Baseline clinical characteristics of the study subjects.

Characteristics	All patients	Healthy controls	Septic shock	P value
Demographics and underlying conditions				
Number of patients	58	28	30	—
Males, number (%)	39(67.2%)	16(57.1%)	27(76.6%)	—
Age (years)	63.5(49.25-72.75)	70.5(63-80.5)	58.5(44.5-67)	0.0199
Hypertension, number (%)	19(32.7%)	8(28.5%)	11(36.6%)	—
Type 2 Diabetes mellitus, number (%)	11(18.9%)	4(6.8%)	7(23.3%)	—
Laboratory value, mean ± SEM				
PCT (μg/L)	—	0.2 ± 0.1	37.6 ± 4.9	<0.0001
CRP (mg/L)	—	8.1 ± 3.7	93.5 ± 5.1	<0.0001
Lactate (mmol/L)	—	0.6 ± 0.1	5.2 ± 1.2	<0.0001
Site of infection, number (%)				
Lung	—	—	14(46.6%)	—
Abdomen	—	—	6(20.0%)	—
Urinary tract	—	—	4(13.3%)	—
Other	—	—	6(20.0%)	—
Origin of the infection, number (%)				
Viral originated	—	—	7(23.3%)	—
Bacterial originated	—	—	18(60.0%)	—
Other	—	—	5(16.7%)	—
Length of stay				
In the ICU (days)	6(0-6)	0	15.5(4.5-24.25)	<0.0001
In the hospital (days)	15(10.75-25.25)	15(10.75-25.25)	26.5(14.5-39.25)	0.0021
APACHE II score	19(13-24)	0	19(15-25)	<0.0001
SOFA score	6(4-10)	0	6.5(4-11)	<0.0001

APACHE II, acute physiology and chronic health evaluation score; SOFA, sequential organ failure assessment score. Data is presented as median (interquartile range); P-value is analyzed by chi-square (gender and site of infection), Kruskal-Wallis test (age) or Mann-Whitney U test (APACHE II score, SOFA score).

## 4 Discussion

Currently, the mortality caused by sepsis and septic shock is still remarkably high. Early detection, suitable categorization, and timely treatment administered during the incipient period of septic shock are vital for decreasing the death rate. Biological information assays allow us to reveal the molecule mechanisms of illness onset and progression, offering a new and valid method to acquire the underlying diagnosis markers and treatment targets that can prevent and treat septic shock. In the present study, we first identified 30 genetic variations from the combined GSE33118 and GSE26440 datasets, and then combined Lasso regression and Boruta feature selection algorithm to screen out six key genes that are involved in septic shock. Subsequently, through intersecting genes between key genes screened from DEGs for septic shock and those identified using WGCNA, we finally identified six new biomarkers of septic shock, namely *CD177*, *CLEC5A*, *CYSTM1*, *MCEMP1*, *MMP8*, and *RGL4*. The PPI networks have proven to be useful in the analysis of many kinds of disease. The STRING online database was used to construct PPI networks in which all protein-coding genes in a genome are grouped and organized. These six biomarkers were also verified by comprehensive bioinformatics analyses of gene expression profiles and a preliminary gene expression assay.

*MMP8* is a Zn^+2^-reliant endopeptidase of the MMP family, which is mainly produced by neutrophilic cells as a proenzyme, and is stimulated by the ROS generated from the stimulated neutrophilic cells; hence*, MMP-8* is vital for acute and persistent inflammatory activities (33, 34). MMP8 is related to the stimulation of proinflammatory cytokine TNF-α (35, 36), which eventually activates inflammatory activities throughout the body. MMP8 participates in the homoeostasis of the exocellular area, the expression of which is mainly realized by monocytes and macrophages. It also participates in the supportive activities of inborn immunity (37). The elevated MMP-8 activities facilitate the etiology of numerous illnesses, such as atherosis, lung fibrosis, and septic disease (38). Pedro Martínez-Paz et al. contrasted septic sufferers separated into septic shock and non-septic shock groups in postsurgical cases (39), then discovered that the septic shock cases registered greater sera MMPs expressing in contrast to the non-septic shock cases and the healthy donors. All discoveries reveal that MMPs can be underlying diagnosis biomarkers of septic shock.

*CD177* serves as a vital neutrophilic cell gene encoding the glycoprotein of the membrane and the expression of such gene is increased during bacterial infections, burns, and pregnancy (40, 41). The identification of *CD177* mRNA concentration has also become a helpful diagnosis method to differentiate these illnesses (42, 43), whereas its function in septic progression is still elusive. The *CD177* gene might serve as an assumed marker or medicine target for septic shock.

Artificial intelligence systems have identified that the novel genetic marker *CLEC5A* helps to predict sepsis severity or mortality; *CLEC5A* had been identified previously and associated with sepsis mortality (44). Recent findings have suggested that CLEC5A (45) might be further implicated in NETosis. The polymorphic nucleus neutrophilic cells, facilitating inflammation representatively, also play roles in immunoregulation functions. A recent study found that CLEC5A, related to dengue, a virus-caused, life-threatening illness (46), is a vital element related to the regulation of the inborn immunoresponse against microbial infectious diseases in mice. This study showed that the expression of CLEC5A was increased in septic shock patients, and that the degree of increase was related to the degree of organic function disorder.

*CYSTM1* is a comparatively elusive gene and the biological information assay displays an effect in resisting various matters involving DNA-injuring agents such as oxidizer, H_2_O_2_ mitomycin C, and the underlying membranous destabilization agent. The variation of the oxidation-reduction potential (ORP) of the membranous substances by CYSTM proteins may also impact the absorption of some substances and enable the quenching of underlying harmful free radicals (47). Thus, consistently across eukaryotes, *CYSTM1* seems to play an effect in stress reaction or resistance, especially against harmful matters (48). The discoveries made in this study indicate that the *CYSTM1* gene might serve as a novel marker for septic shock.

*MCEMP1* is responsible for the encoding of a single-pass trans-membranous protein and participates in the modulation of MC differentiative activities or immunoresponses (49). MCs deteriorate septic disease *via* damaging the phagocyte activity of resident macrophages, hence enabling the proliferative activities of regional and systemic microbe agents (50). Jian-Xin Chen et al. verified that the miR-125-mediation *MCEMP1* suppression could decrease the sera levels of *TNF-α*, *IL-1β*, and *IL-6*, and the programmed cell death facilitated the T leukomonocyte activity, hence attenuating the immune activity of sepsis mice (51). These discoveries reveal that *MCEMP1* can be an underlying diagnosis marker for septic shock.

Yidan Sun et al. evidenced that the reduced expression of *RGL4* was remarkably related to unfavorable prognostic results and immunocyte infiltrative activities in lung adenocarcinoma (LUAD) cases and highlighted the utilization of *RGL4* as a new prediction marker for the prognostic results of LUAD (52). *RGL4* might also be utilized in combination with the immuno-checkpoint method to reveal the advantages of immunity therapy.

In addition, these six potential biomarkers have considerable prospects in other diseases. Reports have shown that *MMP8*, which has a strong correlation with the development of skin cancers (53), particularly breast (54), melanoma (55), and tongue (56), could diminish or accelerate cancer progression *via* its breaking down of the ECM and cleavage of cell adhesion proteins (57). *CD177* modulates the function and homeostasis of tumor-infiltrating regulatory T cells, one of the major immunosuppressive cell types in cancer (58). Tong et al. have reported that *CLEC5A* correlated with immunosuppression in glioma patients (59); meanwhile, *CLEC5A* was identified as an immune-related prognostic gene of ovarian cancer based on the immune microenvironment (39). In addition, bioinformatics analysis demonstrated that *CYSTM1*, a relatively unknown gene, plays a role in stress response (48). Gang Hu et al. were the first to report that the *MCEMP1* gene had a significant association with the prognosis of gastric cancer (60). Moreover, the expression of *RGL4* has been found to be correlated with a variety of tumor-infiltrating immune cells, such as neutrophils, CD8^+^ T cells, and memory B cells (52).

However, the pathways of *CD177*, *CLEC5A*, *CYSTM1*, *MCEMP1*, *MMP8*, and *RGL4* in the development of septic shock remain unclear. Our study suggested that these core genes influenced the progression of septic shock by regulating these pathways, including the reactive oxygen species pathway, hypoxia, PI3K/AKT/mTOR signaling, NFκ-β/TNFα, and IL6/JAK/STAT3. Further relevant experimental assays should be carried out to verify the potential functions of critical genes and pathways in septic shock.

In this study, the CIBERSORT algorithm was used to quantify the abundance of each tumor microenvironment (TME) cell infiltration in the septic shock patient and healthy donors. In total, 22 subpopulations of immune cells, including B cells, plasma cells, T cells, NK cells, monocytes, macrophages, dendritic cells, mast cells, eosinophils, and neutrophils, were evaluated. A significant difference in immune cell infiltration was found between septic shock patients and control participants. Meanwhile, Spearman’s correlation analysis was conducted to evaluate the correlation between related DEGs and each TME infiltration cell type content.

The AUROCs of the six genes in the early diagnosis of septic shock were remarkable, indicating a better predictive efficiency than classical biomarkers such as CRP and PCT. Moreover, the intervention with the signal pathways that are related to these six hub genes may help us to achieve a better treatment response and prognosis for septic shock patients. Our study had inevitable limitations. First, to guarantee universalization and reduce selective bias to a minimum, more verification must be put in place, and larger numbers of septic shock patients and healthy donors should be involved. Second, in this study, our team merely highlighted markers that originated from white blood cells in circulating blood. Circulation markers related to cells from other organs with function disorders, such as tissue macrophages and blood vessel cells, may also participate in an intricate clinical process of septic disease, but these were not investigated in the present study. Last, as we obtained most of our data from specimens of septic shock who were minors and healthy donors minors through the biological information assay, the outcomes cannot be uncritically applied to adult septic cases. Further experimental analysis is still required to validate these findings. In addition, from the vast GEO database, not all septic shock samples could be included; thus, GSE33118, GSE26440, and GSE9692 were chosen in this study, owing to their relatively large sample sizes and representativeness. However, the sample sizes of the three datasets used in this study were small; thus, from a bioinformatics perspective, the results should be further verified, and an increased number of samples should be studied to increase the credibility of results. Our study has shown that in septic patients the mRNA levels of the *CD177*, *CLEC5A*, *CYSTM1*, *MCEMP1*, *MMP8*, and *RGL4* genes were higher in septic patients than in control participants, suggesting the reliability of the preliminary clinical specimen. There is a lack of statistical difference between *CLEC51* and *CYSTM1* among the six genes tested in an mRNA expression analysis. In addition, only the protein levels of *CD177* and *MMP8* were verified owing to the limited experimental time. In a nutshell, a sufficient sample size for validating these hub genes both in terms of their RNA and protein levels is needed for further exploration.

## 5 Conclusion

In the present study, six hub genes with expression differences in septic shock were screened by broadly used biological information assays and WGCNA by integrating multiple datasets. The results revealed six helpful genes that can be used to explore the biomarkers or molecule mechanisms of sepsis shock.

## Data availability statement

The original contributions presented in the study are included in the article/**Supplementary material**. Further inquiries can be directed to the corresponding author.

## Ethics statement

The study (clinical trial registration number: ChiCTR2100053564) was approved by the Institutional Review Board of the First Affiliated Hospital of Wenzhou Medical University, Wenzhou, China, and has been registered at ClinicalTrials.gov. The patients/participants provided their written informed consent to participate in this study.

## Author contributions

CK, YZ, JW, and MQ conceived and designed the experiments. CK, YZ, and MQ executed the experiments and analyzed the samples. CK, YZ, and MQ analyzed the data. CK wrote the first version of the manuscript. JW contributed to the revision of this article. XX contributed significantly to conducting the supplementary experiments required for the revisions. All authors interpreted the data and critically revised the manuscript. All authors contributed to the article and approved the submitted version.

## Supplementary material

Supplementary Figure 1
**(A)** Correlation analysis of immune activation genes and 6 hub genes. **(B)** Correlation analysis of immunosuppression and 6 hub genes. **(C)** Correlation analysis of chemokines and 6 hub genes. **(D)** Correlation analysis of chemokine receptors and 6 hub genes. Asterisk represent significant, red for positive correlation, blue for negative correlation.
