# Supplementary figures and images for: Six potential biomarkers in septic shock: a deep bioinformatics and prospective observational study
Source: Front Immunol. 2023 Jun 8;14:1184700. doi: 10.3389/fimmu.2023.1184700 (PMC10285480; doi:10.3389/fimmu.2023.1184700)

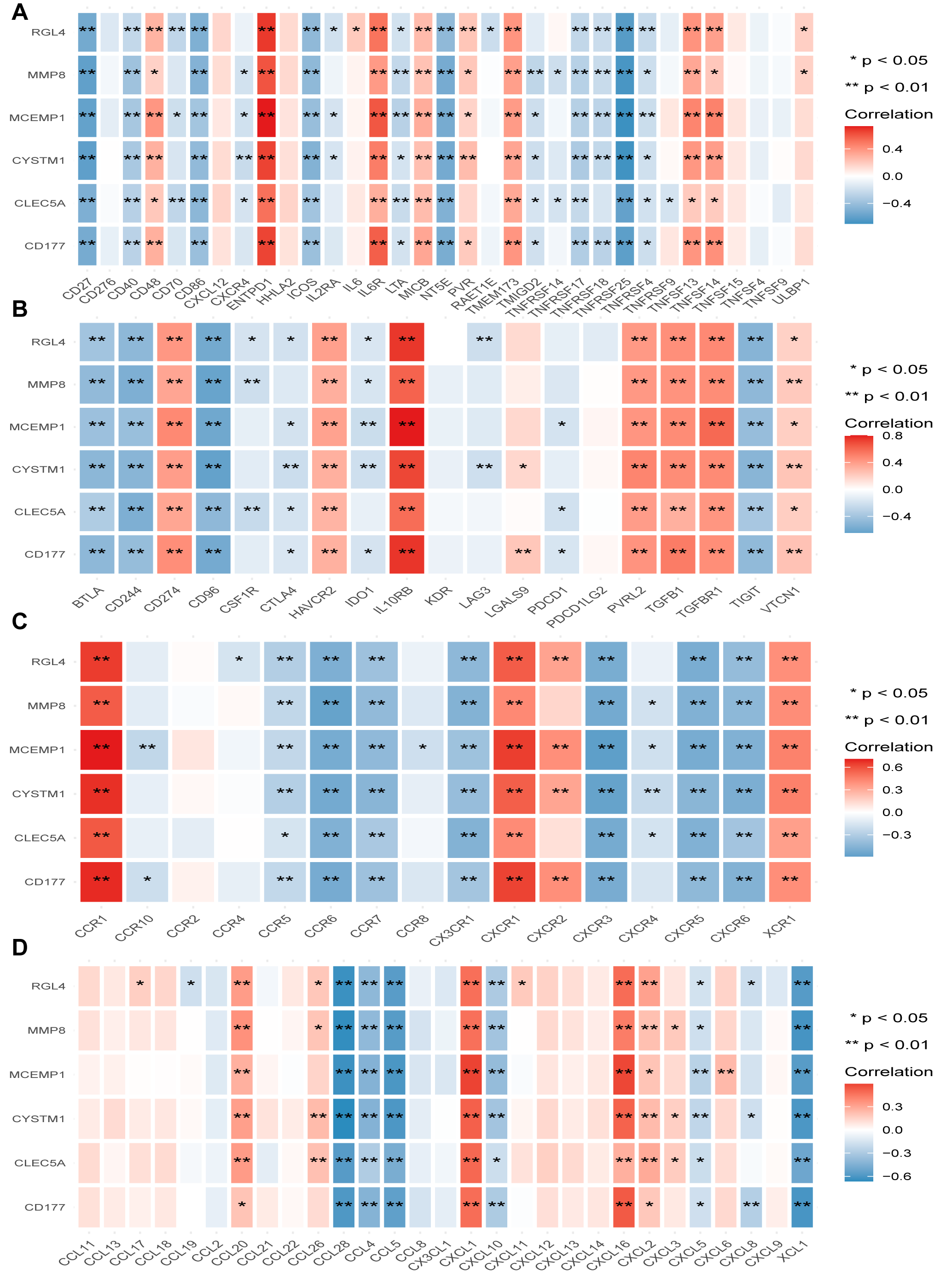

Supplement: Supplementary Figure 1 — (A) Correlation analysis of immune activation genes and 6 hub genes. (B) Correlation analysis of immunosuppression and 6 hub genes. (C) Correlation analysis of chemokines and 6 hub genes. (D) Correlation analysis of chemokine receptors and 6 hub genes. Asterisk represent significant, red for positive correlation, blue for negative correlation. [file Image_1.tif]
