# Supplementary material for: Six potential biomarkers in septic shock: a deep bioinformatics and prospective observational study
Source: Front Immunol. 2023 Jun 8;14:1184700. doi: 10.3389/fimmu.2023.1184700 (PMC10285480; doi:10.3389/fimmu.2023.1184700)
Supplement: Supplementary file 2 [file Table_1.docx]

**Supplementary Table 1. Hub genes identified by Lasso regression**

| **ID** | **logFC** | **AveExpr** | **t** | **P.Value** | **adj.P.Val** | **B** |
| --- | --- | --- | --- | --- | --- | --- |
| **CYSTM1** | 1.195165245 | 2.68641721 | 11.43612416 | 3.20E-22 | 2.51E-18 | 39.7881097 |
| **MCEMP1** | 1.752775752 | 3.423128541 | 11.04140802 | 3.66E-21 | 2.04E-17 | 37.41718842 |
| **MMP8** | 2.984257356 | 4.470139632 | 10.79977703 | 1.62E-20 | 6.36E-17 | 35.96914114 |
| **RGL4** | 1.121878577 | 2.4694539 | 10.44597041 | 1.42E-19 | 2.24E-16 | 33.85537853 |
| **CD177** | 2.421646089 | 4.084602052 | 10.36762246 | 2.30E-19 | 3.38E-16 | 33.38857613 |
| **CLEC5A** | 1.460267358 | 2.6871721 | 9.843242553 | 5.61E-18 | 4.55E-15 | 30.27936902 |
