# Supplementary material for: Six potential biomarkers in septic shock: a deep bioinformatics and prospective observational study
Source: Front Immunol. 2023 Jun 8;14:1184700. doi: 10.3389/fimmu.2023.1184700 (PMC10285480; doi:10.3389/fimmu.2023.1184700)
Supplement: Supplementary file 3 [file Table_2.docx]

**Supplementary Table 2. Hub genes identified by Bruta feature selection algorithm**

| **ID** | **logFC** | **AveExpr** | **t** | **P.Value** | **adj.P.Val** | **B** |
| --- | --- | --- | --- | --- | --- | --- |
| **CYSTM1** | 1.195165245 | 2.68641721 | 11.43612416 | 3.20E-22 | 2.51E-18 | 39.7881097 |
| **MCEMP1** | 1.752775752 | 3.423128541 | 11.04140802 | 3.66E-21 | 2.04E-17 | 37.41718842 |
| **GYG1** | 1.094059454 | 2.494495832 | 11.0139518 | 4.34E-21 | 2.04E-17 | 37.25249822 |
| **MMP8** | 2.984257356 | 4.470139632 | 10.79977703 | 1.62E-20 | 6.36E-17 | 35.96914114 |
| **GPR84** | 1.457976648 | 2.747549891 | 10.59101105 | 5.85E-20 | 1.38E-16 | 34.72082926 |
| **RGL4** | 1.121878577 | 2.4694539 | 10.44597041 | 1.42E-19 | 2.24E-16 | 33.85537853 |
| **CD177** | 2.421646089 | 4.084602052 | 10.36762246 | 2.30E-19 | 3.38E-16 | 33.38857613 |
| **RETN** | 1.733100079 | 3.073876108 | 10.27117765 | 4.15E-19 | 5.42E-16 | 32.81467799 |
| **HP** | 1.690370736 | 3.279075279 | 10.16543427 | 7.91E-19 | 9.79E-16 | 32.18643375 |
| **BMX** | 1.175969956 | 2.509557546 | 9.885363617 | 4.34E-18 | 3.65E-15 | 30.52802268 |
| **CLEC5A** | 1.460267358 | 2.6871721 | 9.843242553 | 5.61E-18 | 4.55E-15 | 30.27936902 |
| **CA4** | 1.034255746 | 2.398455713 | 9.70915984 | 1.26E-17 | 8.25E-15 | 29.48928841 |
| **IL1R2** | 1.580882305 | 3.094504657 | 9.644226026 | 1.87E-17 | 1.16E-14 | 29.10749733 |
| **S100A12** | 1.112287067 | 2.734257997 | 9.586847512 | 2.64E-17 | 1.59E-14 | 28.77060173 |
| **ARG1** | 1.157536493 | 2.453227531 | 9.497758571 | 4.52E-17 | 2.47E-14 | 28.24843201 |
| **ANKRD22** | 1.411189545 | 2.803402392 | 9.084692012 | 5.35E-16 | 2.00E-13 | 25.84331727 |
| **ANXA3** | 1.571831895 | 3.449906134 | 8.92198928 | 1.40E-15 | 4.79E-13 | 24.90398112 |
| **CLEC4D** | 1.136212622 | 2.576669577 | 8.861196482 | 2.01E-15 | 6.39E-13 | 24.55428238 |
| **OLFM4** | 2.465689257 | 3.742248418 | 8.171390163 | 1.12E-13 | 1.91E-11 | 20.64169107 |
| **LCN2** | 1.507361083 | 2.914099654 | 7.749891698 | 1.24E-12 | 1.50E-10 | 18.30923042 |
| **TDRD9** | 1.107138219 | 2.517406783 | 7.743491084 | 1.29E-12 | 1.51E-10 | 18.27419955 |
| **LTF** | 1.512595365 | 3.00423143 | 7.610546827 | 2.71E-12 | 2.82E-10 | 17.54938525 |
| **OLAH** | 1.547806036 | 2.874495704 | 6.74610758 | 3.02E-10 | 1.38E-08 | 12.98128904 |
| **CRISP3** | 1.097507931 | 2.542567103 | 5.677627423 | 6.77E-08 | 1.34E-06 | 7.758501072 |
